# Berberine enhances cisplatin efficacy in ehrlich ascites carcinoma via modulation of apoptotic pathway and efferocytosis

**DOI:** 10.1038/s41598-026-49296-3

**Published:** 2026-04-28

**Authors:** Maha M. Salem, Safia M. Dawod, Tarek M. Mohamed, Wafaa M. Ibrahim, Amira T. Khattab

**Affiliations:** 1 Biochemistry Division, Chemistry Department, Faculty of Science, Tanta University, Tanta, 31257 Egypt; 2 Medical Biochemistry Department, Faculty of Medicine, Tanta University, Tanta, Egypt; 3 Biochemistry Department, Faculty of Science, Damanhour University, Damanhour, 22514 Egypt

**Keywords:** Berberine, PI3K/Akt pathway, Efferocytosis, Apoptosis, Ehrlich Ascites Carcinoma, Cisplatin, Phagocytosis, Cancer, Drug discovery, Oncology

## Abstract

Berberine, a bio-alkaloid from medicinal plants, shows therapeutic potential against various ailments, including cancer. This study evaluated berberine’s effects on the PI3K/Akt pathway and efferocytosis in an adenocarcinoma model to reduce cisplatin chemotherapy side effects and Ehrlich ascites carcinoma (EAC) proliferation. Eighty Swiss albino mice were divided into eight groups: control, berberine, cisplatin, cisplatin & berberine, EAC, EAC & berberine, EAC & cisplatin, and EAC & cisplatin & berberine. Tumor response, weight change, cell count, survival analysis, biochemical analysis, molecular studies, and histopathological assessment were performed. Results showed berberine enhanced cisplatin’s antitumor efficacy in EAC-bearing mice by reducing tumor volume and cell counts. Berberine ameliorated cisplatin’s hepato-renal toxicity and downregulated Akt1, Axl, Mertk, and Gas6 gene expression. Histopathological analysis showed berberine’s ability to recover cisplatin’s lethal effects on liver tissue. In conclusion, berberine showed promising effects on the PI3K/Akt pathway and efferocytosis, reducing cisplatin’s side effects and EAC proliferation.

## Introduction

Natural products have long been recognized as a valuable source of bioactive compounds with significant health-promoting properties^1^. They have been widely utilized in both traditional and modern medicine due to their diverse pharmacological activities, including antioxidant, anti-inflammatory, and anticancer effects^2^. Various classes of phytochemicals, such as alkaloids, flavonoids, and polyphenols, have demonstrated promising therapeutic potential in the prevention and treatment of numerous diseases, including cancer management and modulation of multiple cellular pathways^3^.

Cancer is a complex disease characterized by uncontrolled cell growth and the potential to spread throughout the body, with approximately ten million new cases reported annually worldwide^4,5^. Both endogenous factors, such as genetic predisposition and inherited mutations, and exogenous factors, including diet, chemicals, and radiation, contribute to cancer development^6^. The phosphatidylinositol-3-kinase (PI3K/Akt) pathway, frequently activated in human cancers, plays a crucial role in regulating cellular processes like proliferation, cell cycle, and apoptosis^7^. Consequently, significant efforts have been directed towards developing new drugs targeting this pathway^8^. While chemotherapy remains a highly successful treatment option, especially for metastatic cancers, it often causes severe adverse effects and may lead to drug resistance^9,10^. This has prompted a growing interest in natural compounds as alternative or complementary anticancer agents with lower toxicity and fewer side effects^11^. Berberine (BBR), a bioalkaloid compound isolated from various medicinal plants, has shown broad-spectrum therapeutic potential against ailments including cancer^12^. BBR inhibits proliferation and induces apoptosis and cell cycle arrest in different cancers^13–16^. In addition, emerging evidence suggests that cellular clearance mechanisms such as efferocytosis play a critical role in cancer progression and tumor microenvironment regulation. Efferocytosis, the process by which apoptotic cells are cleared by phagocytes, is essential for maintaining tissue homeostasis and resolving inflammation^17,18^. This process involves four stages: the “find me” stage, the “eat me” stage, endocytosis, and post-phagocytosis^19–21^. Recent studies have demonstrated that berberine targets multiple oncogenic pathways, including PI3K/Akt/mTOR signalling, and enhances the sensitivity of cancer cells to chemotherapeutic agents^22,23^.

The present study aims to evaluate the biochemical and molecular effects of berberine on the PI3K/Akt signalling pathway and investigate its impact on efferocytosis in an adenocarcinoma model. This research seeks to reduce the side effects of cisplatin chemotherapy and inhibit EAC proliferation.

## Materials and methods

### Chemicals and drugs

Cisplatin (cis-diamminedichloroplatinum II) (50 mg/50 mL) was purchased by EIMC united pharmaceuticals, Egypt. Berberine chloride was purchased from (Sigma-Aldrich., USA). Bio Diagnostic company (Egypt) supplied L-malondialdehyde (MDA) (cat. no. MD 25 29), catalase (CAT) (cat. no. CA 25 17), and glutathione peroxidase (GPx) (cat. no. GP 2524) kits. Vitro scient-Diagnostics (Egypt) provided total protein (TP) (cat. no. TP 20 20), albumin (Alb) (cat. no. AB 10 10), creatinine (Create) (cat. no. CR 12 50), urea (cat. no. UR 21 10), alkaline phosphatase (ALP) (cat. no. AP 10 20), alanine transaminase (ALT) (cat. no. AL 10 31), and aspartate transaminase (AST) (cat. no. AS 10 61). RNA extraction (‘Cat no. # K0731), and Reverse transcription (Cat. no. EP0451) kits purchased from Thermo Scientific, (Fermentas). ELISA kits for (CRT) (Cat. No. 201-02-1232) and CD47 (Cat. No. 201-02-1682), (SunRed, China) Akt1, Axl, Mertk and Gas6 genes with S18 as a housekeeping genes primers were from Biotech. Company, (Egypt). All other chemicals used were high analytical grade.

### Experimental animals and ethical approvements

Eighty Swiss albino mice, weighing 20–25 g, were obtained from the National Cancer Institute, Cairo University. The animals were housed in steel mesh cages under controlled conditions: 12:12 h light-dark cycle and temperature of 20–22 °C. They were maintained on a commercial standard pellet diet and drinking water for a 2-week acclimatization period. The study was conducted in accordance with the guidelines for the care and use of laboratory animals approved by the Research Ethical Committee (Faculty of Science, Tanta University, Egypt), which aligns with the National Institutes of Health requirements (NIH publications no. 8023, revised 1978). The ethical approval committee number is [**IACUC-SCI-TU-0341**].

### Ehrlich ascites carcinoma cells and tumor inoculation

**Fig. 1:**
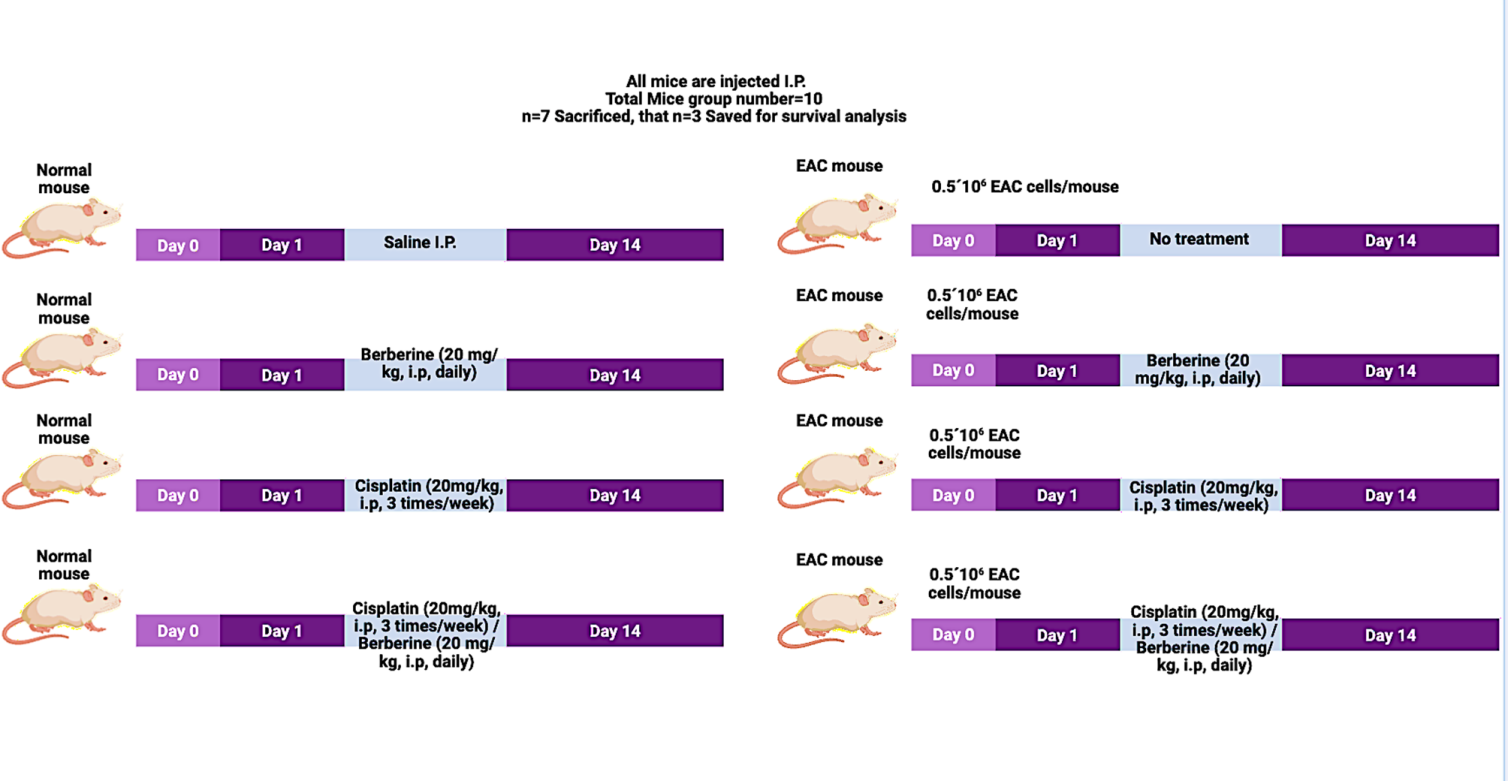
Schematic representation of the experimental design and treatment protocol. Mice were divided into eight groups (*n* = 10 per group), including normal and Ehrlich ascites carcinoma (EAC)-bearing groups. On day 0, EAC was induced in groups V–VIII by intraperitoneal injection of 0.5 × 10⁶ EAC cells/mouse. Treatment started on day 1 and continued for 14 days. Normal groups received either saline, berberine (20 mg/kg, *i.p*., daily), cisplatin (20 mg/kg, *i.p*., three times per week), or their combination. EAC-bearing groups were treated with the same regimens as their corresponding normal groups. The timeline illustrates the sequence of EAC inoculation, treatment initiation, and duration. That, EAC, Ehrlich ascites carcinoma; *i.p*., intraperitoneal. Note That: “n = 7 sacrificed at day 14” and “n = 7 followed for survival”.

Ehrlich ascites carcinoma (EAC) cells were initially obtained from the National Institute of Cancer (Cairo, Egypt). EAC cells were collected and diluted with sterile normal saline (0.9% NaCl)^24^. Viable and dead EAC cells were counted using trypan blue method^25^. To develop tumor-bearing mice EAC cells number were adjusted at 0.5 × 10^6^ cells/mice in 0.1 mL sterile saline for intraperitoneal (*i.p.*) inoculation into healthy Swiss albino mice as illustrated in Fig. 1^26^.

### Experimental design

Eight groups of mice (*n* = 10 per group) were included in this study. Each group initially consisted of 10 mice. Seven mice per group (*n* = 7) were used for biochemical, molecular, and histopathological analyses, while the remaining three mice (*n* = 3) were reserved for survival analysis. Group I (Normal control): mice received normal saline intraperitoneally (*i.p*.). Group II: mice received berberine (20 mg/kg, *i.p*., once daily)^27^. Group III: mice received cisplatin (20 mg/kg, *i.p*., three times per week)^28^. Group IV: mice received a combination of cisplatin and berberine at the same previously mentioned doses and schedules. Group V (EAC control): mice were intraperitoneally inoculated with 0.5 × 10⁶ Ehrlich ascites carcinoma (EAC) cells per mouse on day 0 and received no further treatment. Group VI: EAC-bearing mice received berberine (20 mg/kg, *i.p*., once daily) starting from day 1 for 14 consecutive days. Group VII: EAC-bearing mice received cisplatin (20 mg/kg, *i.p*., three times per week) starting from day 1 for 14 days. Group VIII: EAC-bearing mice received a combination of cisplatin and berberine using the same dosing regimens for 14 days. All treatments were initiated one day after EAC inoculation (day 1) and continued for 14 days. The overall experimental timeline and treatment schedule are illustrated in Fig. 1.

### Sampling

On day 14, mice were euthanized by overdose of sodium pentobarbital (300 mg/kg, intraperitoneal injection) under deep anesthesia^29^. EAC cells were withdrawed from the peritoneal cavity for counting using the trypan blue method^25^. Ascetic fluid volume was measured using a graduated centrifuge tube. Serum collected, and tissue samples from the liver were divided into two parts: first one was fixed in 10% neutral buffered formalin for histological analysis, second part of tissues were preserved at − 20 °C for biochemical analysis.

### Body weight change

Mice weighed at the beginning (initial body weight) and at the end of the experiment (final body weight), the body weight change calculated according to^30,31^.

### Cell count using trypan blue

To assess cell count and viability, EAC cells were stained with 0.4% trypan blue prepared in 1× phosphate buffer saline (PBS)^25^, and counted in a hemocytometer according to the following formula:

$$\:\mathrm{N}\mathrm{u}\mathrm{m}\mathrm{b}\mathrm{e}\mathrm{r}\:\mathrm{o}\mathrm{f}\:viable\:cells\%=\:\frac{\left(total\:number\:of\:cells-number\:of\:trypan\:blue\:positive\:cells\right)}{Total\:number\:of\:cells}\times\:100$$

### Survival analysis

For survival analysis, a subset of mice (*n* = 3 per group) was reserved from the beginning of the experiment and was not subjected to euthanasia at the end of the 14-day treatment period. The remaining mice (*n* = 7 per group) were sacrificed after 14 days for biochemical and histological investigations. The reserved mice were monitored daily for survival until spontaneous death occurred, which was considered the experimental endpoint. Mean survival time (MST), increased life span (ILS%), MST fold change, and tumor growth inhibition percentage (T/C%) were calculated as follows^32^.

$$\:MST=\frac{1st\:day\:of\:death+last\:day\:of\:death}{2}$$

$$\:ILS\left(\%\right)=\:\left\{\left(\frac{MST\:of\:treated\:group}{MST\:of\:control\:group}\right)-1\right\}\times\:100$$

$$\:T/C\:\left(\%\right)=\:\left(\raisebox{1ex}{$MST\:of\:treated\:group$}\!\left/\:\!\raisebox{-1ex}{$MST\:of\:control\:group$}\right.\right)$$

### Biochemical analysis

#### Serum and tissue analysis

ALT, AST, and ALP activity were assessed according to^33,34^. Creatinine and urea concentrations followed^35,36^. Serum TP and Alb levels were determined ^37,38^. Hepatic and renal levels of MDA, CAT, and GPx were determined calorimetrically ^39,40^.

#### Efferocytosis assessments

ELISA assay was performed for assessment of calreticulin (CRT) and CD47 according to the manufacturer’s protocol.

### Molecular investigations

#### Apoptotic profile of EAC cells

EAC cells from treated mice were washed with PBS, measured for density, and re-suspended in annexin-binding buffer to 1 × 10^6^ cells/ml. The cell suspension (100 µl) was placed in Eppendorf tubes with annexin V-FITC (5 µl) and PI working solution (1 µl, 100 µg/ml). After 15-minute room temperature incubation, 400 µl of annexin-binding solution was added, mixed, and kept on ice. Cells were analyzed by flow cytometry^41^.

#### Cell cycle arrest of EAC cells

EAC cells from treated mice were isolated at 2 × 10^6 cells/ml, washed with PBS, and fixed in 70% ethanol at 4 °C overnight. Cells were suspended in PI/Triton X 100 solution (1000 µl of 0.1% Triton, 40 µl of PI, 20 µl of RNase) for 30 min at 37 °C. Cell cycle distribution was analyzed using flow cytometry (BD FACSCanto II) with BD FACS Diva software^42^.

#### Gene expression by q-RT-PCR

Total RNA was extracted from fresh EAC cells using the Gene JET RNA Purification Kit. The purity, quality, and concentration of the extracted RNA were determined using a Nano Drop Spectrophotometer (Analytik Jena model ScanDrop, Germany). cDNA synthesis was performed using the RevertAid First Strand cDNA Synthesis kit. Quantitative real-time PCR (qRT-PCR) with SYBR Green was used to measure the expression of Akt1, Axl, Mertk and Gas6 genes with S18 as an internal reference. The PCR reactions were performed in a Step One Plus real-time thermal cycler (Applied Biosystems 2720, Life technology, USA) with the PCR primers designed using IDT online software specified in Table 1. Assessment of 2^−ΔΔ Ct^ determined the fold change in gene expression relative to the house keeping gene.

**Table 1:** Primer sequence used in qRT-PCR.

Genes	Primers sequence		References
	Forward primer (5՝−3՝)	Reverse primer (5՝−3՝)	
Akt1	GGTCAGCAAGGAAGTGGAAGT	GCTGGAGAAGCTGGATGAAC	^43^
Axl	ATGAGCGGAGCGGAGCGGAGC	CTAGGCGCGCTGAGGCGAA	^44^
Gas 6	TGGCAGCATGGATGAAGGAAG	TGGGAGGTCAGGCTTTGTTCT	^45^
Mert-K	ATGCACCGCCACATCTACAG	TCAGGCAGCTGGTAAGACTG	^45^
S18	GGTAACCCGTTGAACCCCATT	CAAGCTTATGACCCGCACTT	^46^

#### Histopathological assessment

Liver sections were fixed in 10% formalin, then dehydrated using ethanol, cleared in xylene and then embedded in paraffin wax, and sectioned into 4 μm, and then stained with Hematoxylin and Eosin^47^. All stained slides were viewed and captured by Olympus microscope digital camera (Cannon 620). Brightness, contrast, and analysis of the images were adjusted using Adobe Photoshop software (version 4.0.1; Adobe Systems, Mountain View, CA).

#### Statistical analysis

The results were expressed as mean ± SE. Statistical analyses were conducted using GraphPad Prism version 6. To determine statistical significance, one-way analyses of variance (ANOVA) were utilized. A *p*-value of ≤ 0.05 was considered to indicate statistical significance.

## Results

### Berberine/Cisplatin combination decreased body weight in EAC mice

**Fig. 2:**
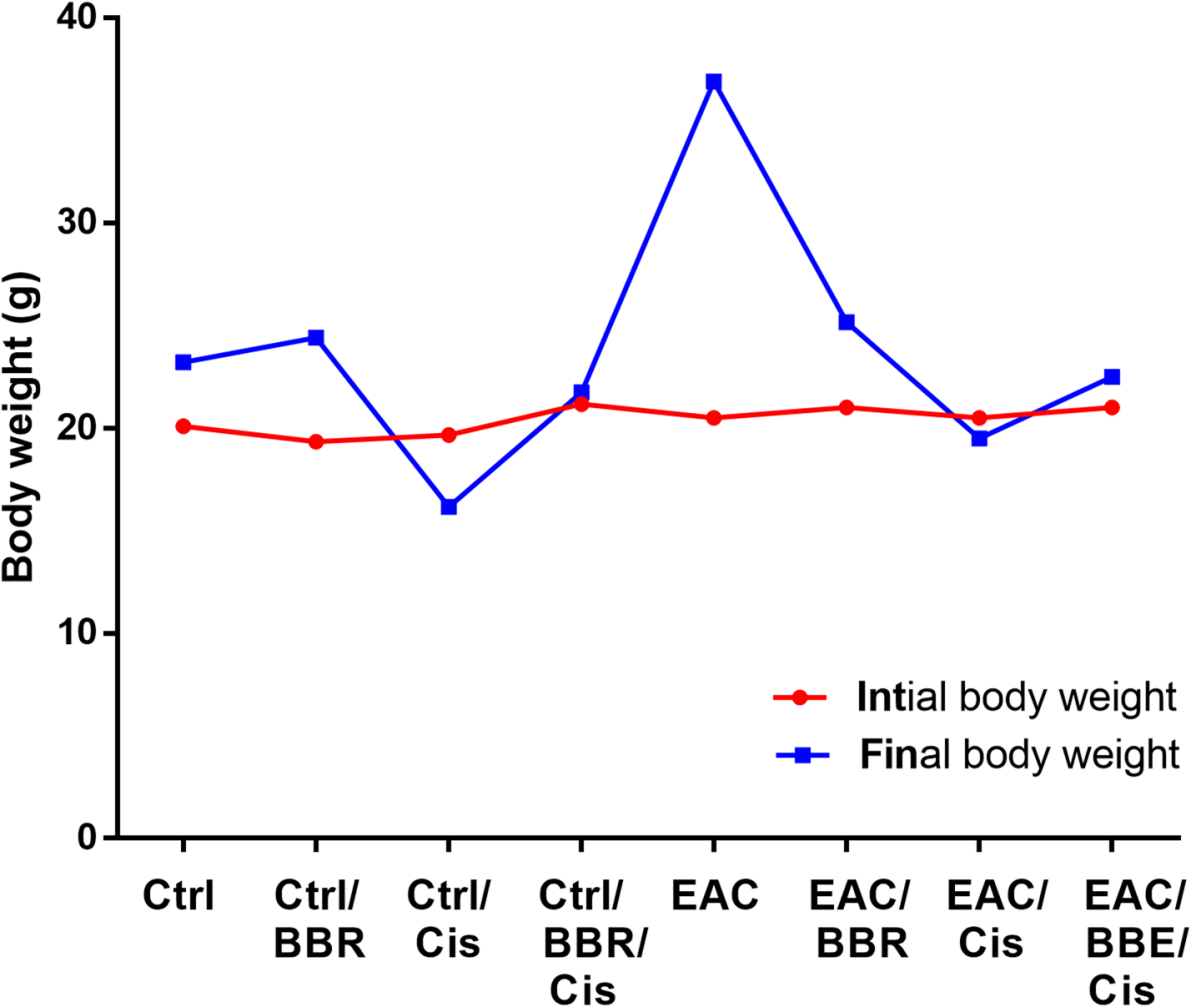
Initial and final body weight of all groups. Results were expressed as mean ± SE, (*n* = 7). *p* < 0.05 is considered significant.

There was no significant difference in initial body weight between all groups. Final body weight of non-treated EAC mice (*gpV*) was (36.9 ± 4.0 g, *n* = 7) and it was significantly increased (*p* < 0.0001) than normal control mice (*gpI*) (23.2 ± 0.33 g, *n* = 7). Treatment of EAC mice with BBR and Cis alone or in combination resulted in a significant decrease in body weight with (*p* < 0.001) and (*p* < 0.0001) or (*p* < 0.0001), respectively, as compared with non-treated EAC-bearing mice (Fig. 2).

### Berberine enhance the antitumor efficacy of cisplatin in EAC-bearing mice

**Fig. 3:**
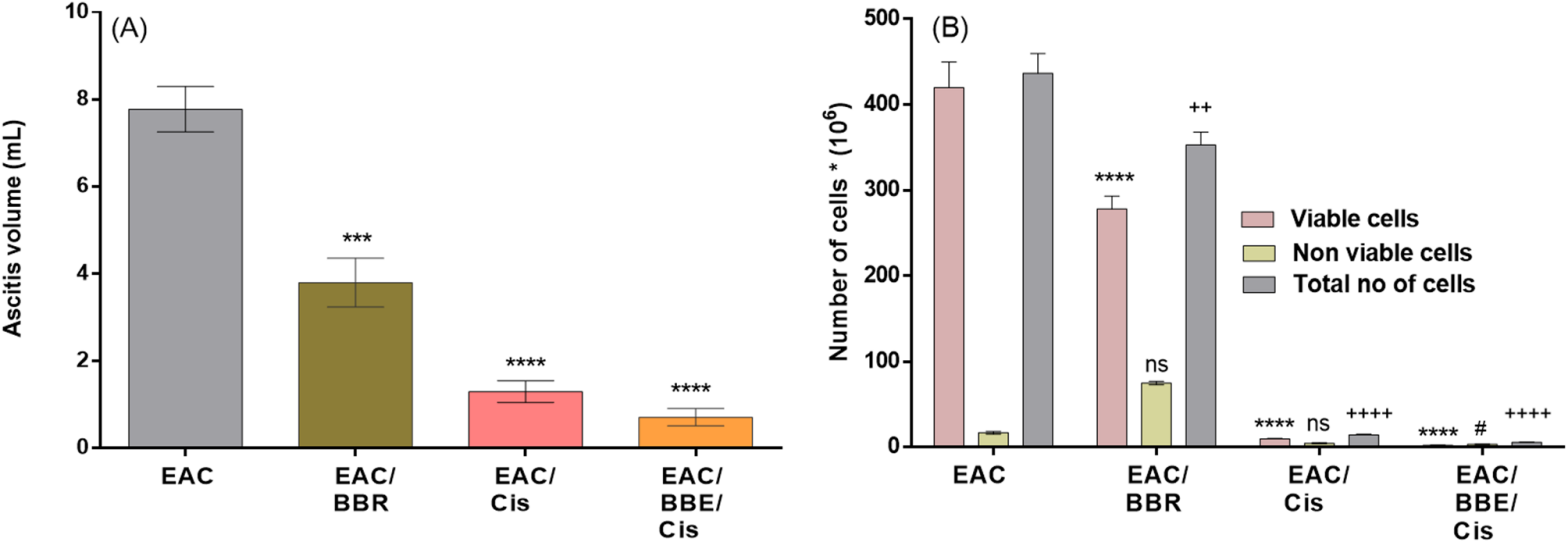
EAC cell count **(A)**, and ascitic volume **(B).** Results were expressed as mean ± SE, (*n* = 7). *p* < 0.05 is considered significant, where ^*^ compared with EAC group, viable cell count, ^#^ compared to non-viable cell count, and ^+^ compared with total cell count.

Treatment of EAC-bearing mice with the BBR (*gp VI*) led to a significant reduction in values of the total tumor volume (3.8 ± 0.5) (*p* ≤ 0.001), total tumor cells count (352.7 ± 15) (*p* ≤ 0.01), and live tumor cells count (278 ± 15) (*p* ≤ 0.01) and elevating in dead cells which counted (74.75 ± 2.1) (*p* ≤ 0.01) when compared with the corresponding values in the EAC-bearing mice (*gp V*) that are equal to (7.7 ± 0.5), (436.8 ± 23), (420 ± 30) and (16.8 ± 1.5) respectively. While in EAC-bearing mice that treated with Cis (*gp VII*), the total tumor volume, total tumor cells count, and live cells tumor count were reduced more than their corresponding values in the group of EAC-bearing mice that treated with berberine alone with values equal to ((1.3 ± 0.24) (*p* ≤ 0.0001), (14.14 ± 0.98) (*p* ≤ 0.0001) and (9.5 ± 0.65) (*p* ≤ 0.0001) respectively. Co-treatment BBR/Cis (*gp VIII*) increased the reduction significantly in the total tumor volume (0.7 ± 0.19) (*p* ≤ 0.05), total tumor cells count (5.6 ± 0.21) (*p* ≤ 0.01), and viable tumor count (2.27 ± 0.09) (*p* ≤ 0.01) when compared to EAC/cis mice (*gp VII*) as shown in (Fig. 3A, B).

### Berberine/Cis combination increased MST, %ILS, and T/C % of EAC-bearing mice

**Fig. 4:**
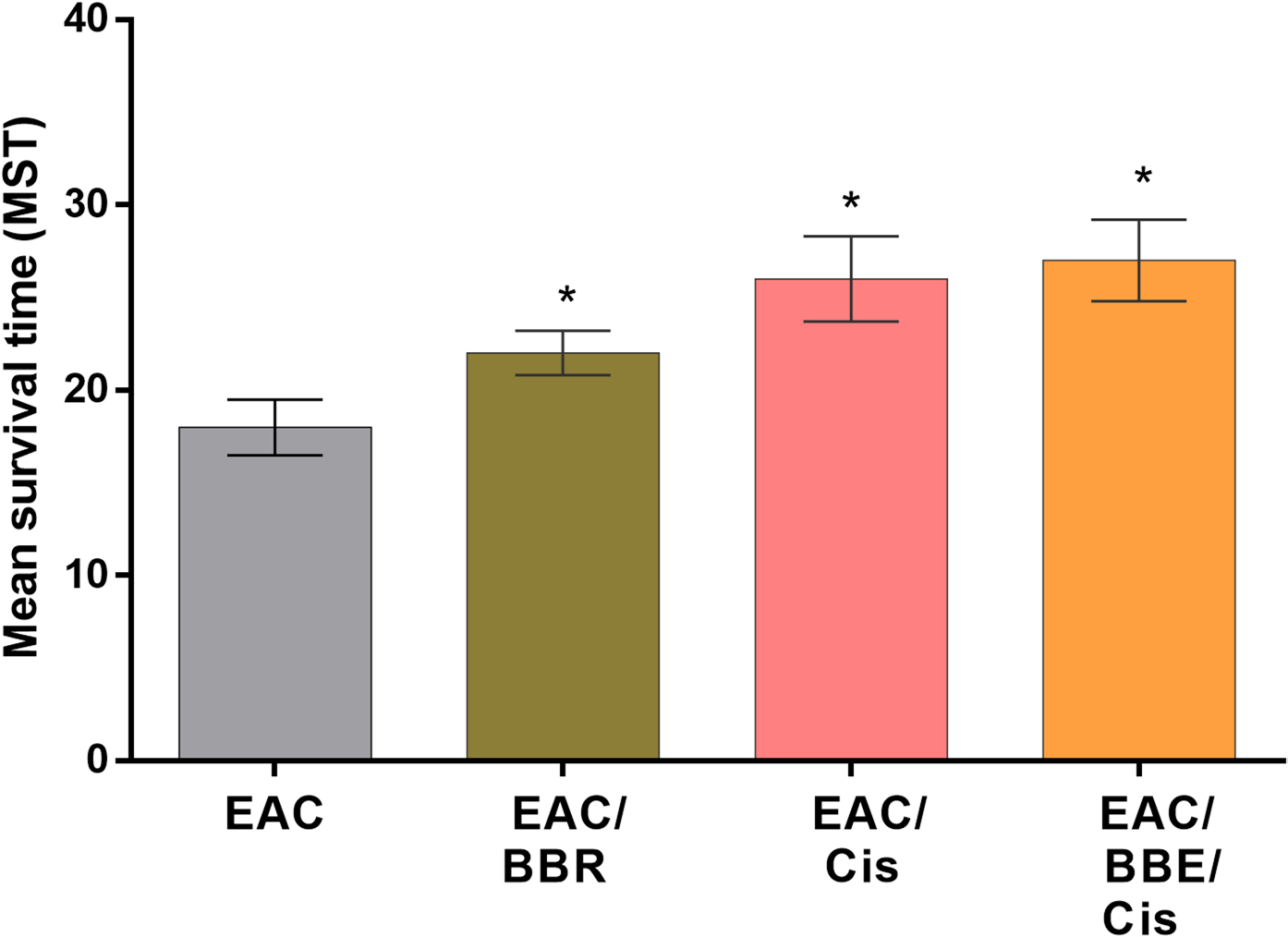
The mean survival time of all groups. Data are represented as mean ± SE, *P* < 0.05 (*n* = 3), where ^*^ compared with EAC non-treated group.

The data obtained revealed the effect of BBR, Cis alone and in combination (BBR/Cis) on MST, %ILS and T/C%. The values of all groups increased relative to that of EAC non-treated group. The highest values for MST, %ILS and T/C% are given in the combined treatment Cis with BBR as 30 days, 66.6% and 166% respectively as shown in Table 2: Fig. 4.

**Table 2:** %ILS and T/C % in different groups.

Groups	% ILS	T/C%
EAC	0	0
EAC/BBR	22.2	122
EAC/Cis	44.4	144
EAC/BBR/Cis	66.6	166

Results were expressed as (mean ± SE, *n* = 3), *p* < 0.05 is considered significant, where ^*^ showed significance vs. EAC non-treated group.

### Berberine enhanced the liver and kidney functions of cisplatin in EAC-bearing mice

Regarding serum liver function tests, EAC and Cis control groups showed a significant elevation in serum aminotransferases while they demonstrated a significant reduction in albumin and total protein serum level as compared to control group. Additionally, there is no significant difference between BBR and BBR/Cis control groups versus the control group concerning all liver function tests. Apart from AST which illustrated significant reduction in EAC/BBR group versus EAC non treated group, there is no significant change in ALT, albumin, and total protein serum levels. In addition, EAC/BBR/Cis group exhibited significant decrease in ALT and AST serum level while they proved significant increase in albumin and total protein serum level as compared to the EAC non treated group. Furthermore, EAC/Cis showed no significant differences in all liver function tests in comparison with EAC non treated group.

Concerning renal function test, EAC group as well as Cis control group demonstrated a significant increase in urea and creatinine serum level compared with normal control. It is also worth noting that BBR control, and BBR/Cis control groups had no significant differences in renal function test as compared to normal control. Furthermore EAC/BBR/Cis group demonstrated a significant lessening in urea and creatinine serum level against the EAC non treated groups. Moreover, EAC/BBR, and EAC/Cis groups exhibited a significant difference in renal function test as compared to the EAC non treated group (Table 3).

**Table 3:** Liver and Kidney functions in different groups under the study.

Groups	ALT (U/L)	AST (U/L)	Alb (g/dL)	T.*P* (g/dL)	Urea (mg/dl)	Creatinine (mg/dl)
Ctrl	43 ± 3.5	71.5 ± 4.4	3.3 ± 0.09	7.1 ± 0.22	57.6 ± 5.1	0.42 ± 0.02
Ctrl/BBR	40 ± 2.9 n.s	73.0 ± 6.9 n.s	3.5 ± 0.1 n.s	7.3 ± 0.23 n.s	55 ± 3.4 n.s	0.5 ± 0.025 n.s
Ctrl/Cis	98 ± 4.5 ^+^	169.4 ± 9.8^+^	2.18 ± 0.2^+^	5.9 ± 0.2 ^+^	95.5 ± 4.5 ^+^	1.3 ± 0.07^+^
Ctrl/BBR/Cis	51 ± 3.2 n.s	87.1 ± 7.1 n.s	2.9 ± 0.08 n.s	6.8 ± 0.21 n.s	72.5 ± 4.6 n.s	0.6 ± 0.039 n.s
EAC	75 ± 6.0 ^+^	212 ± 13.9 ^+^	2.1 ± 0.14 ^+^	4.8 ± 0.13 ^+^	98 ± 3.8 ^+^	1.0 ± 0.07 ^+^
EAC/BBR	69 ± 5.1 n.s	150 ± 7.0^*^	2.2 ± 0.2 n.s	5.0 ± 0.14 n.s	90 ± 4.2 n.s	0.75 ± 0.03^*^
EAC/Cis	79 ± 4.3 n.s	190 ± 6.6 n.s	2.0 ± 0.17 n.s	4.7 ± 0.12 n.s	84.7 ± 5.4 n.s	0.85 ± 0.04^*^
EAC/BBR/Cis	58.9 ± 6.9 ^*^	88.3 ± 30.2 ^*^	2.7 ± 0.22 ^*^	5.9 ± 0.11^*^	67.4 ± 10.2^*^	0.62 ± 0.06^*^

Results were expressed as (mean ± SE, *n* = 7), *p* < 0.05 is considered significant, where n.s means non-significant, ^*+*^ showed significance vs. Normal control group and ^*^ showed significance vs. EAC non-treated group.

### Berberine ameliorate the hepato-renal toxicity of cisplatin in EAC-bearing mice

Both hepatic and renal MDA level were significantly elevated in EAC and Cis control group with, while BBR control and BBR/Cis control groups displayed non-significant differences as compared to normal control group. Also, EAC/BBR group demonstrated a statistically significant reduction in both hepatic and renal MDA levels as compared to the EAC non treated group. On the other hand, EAC/BBE/Cis group showed a statistically significant elevation in both hepatic and renal MDA level as compared to the EAC non treated group. Furthermore, EAC/Cis groups displayed a statistically significant decrease in hepatic MDA level without causing any significant changes in renal MDA level in comparison with EAC non treated group.

**Fig. 5:**
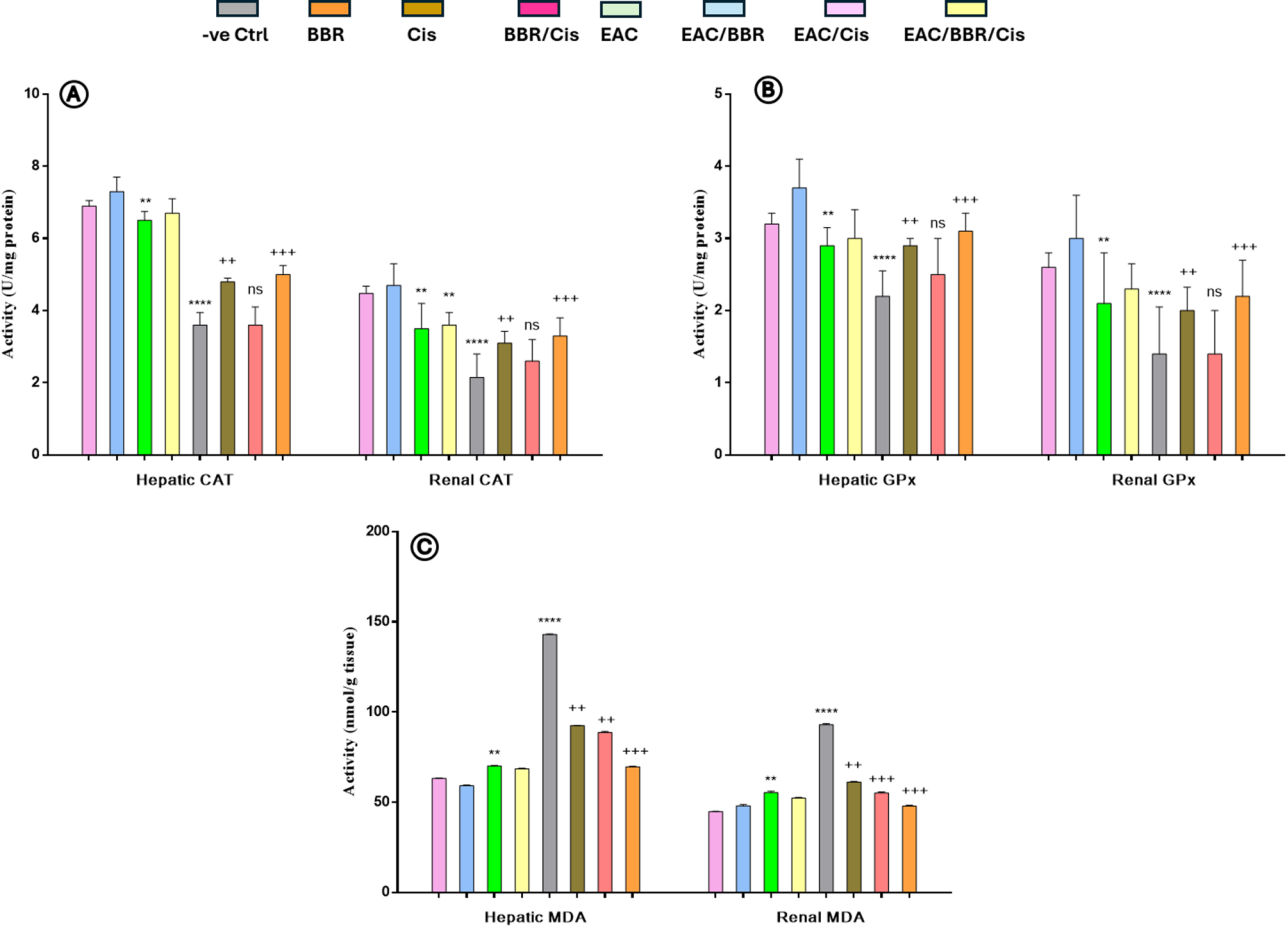
Hepatic and renal catalase (CAT) **(A)**, glutathione peroxidase (GPx) **(B)** and Malonaldehyde (MDA) **(C)** in all groups. Results were expressed as (mean ± SE, *n* = 7), *p* < 0.05 is considered significant, where n.s means non-significant, ^*+*^ showed significance vs. Normal control group and ^*^ showed significance vs. EAC non-treated group.

As compared to the control group; BBR control group had no significant differences in either hepatic or renal antioxidants. On the opposite Cis control and EAC groups showed a significant decline in all hepatic and renal antioxidants as compared to the control group. BBR/Cis control group displayed a significant reduction (*p* < 0.05) in renal catalase, while it displayed non-significant differences in hepatic catalase and both hepatic and renal GPx level in comparison with the control group. Hepatic catalase showed significant reduction in EAC/BBR/Cis (*p* < 0.05) without displaying any significant changes in EAC/Cis group compared to EAC non treated group and significant EAC/BBR group. On the other hand, renal catalase showed significant elevation (*p* < 0.05) in EAC/BBR group and significant reduction in EAC/BBR/Cis groups (*p* < 0.05), while displayed non-significant difference in EAC/Cis compared with EAC non treated group. Furthermore, EAC/BBR group showed significant increase (*p* < 0.05) in both renal and hepatic GPx. Morover hepatic GPx displayed significant reduction in EAC/BBR/Cis group without any significance difference in EAC/Cis groups versus EAC non-treated group, while renal GPx showed significant reduction in EAC/BBR/Cis groups and non-significant change in EAC/Cis groups compared to EAC non-treated group as shown in (Fig. 5).

### Berberine evaluated efferocytosis biomarker proteins of cisplatin in EAC-bearing mice

**Fig. 6:**
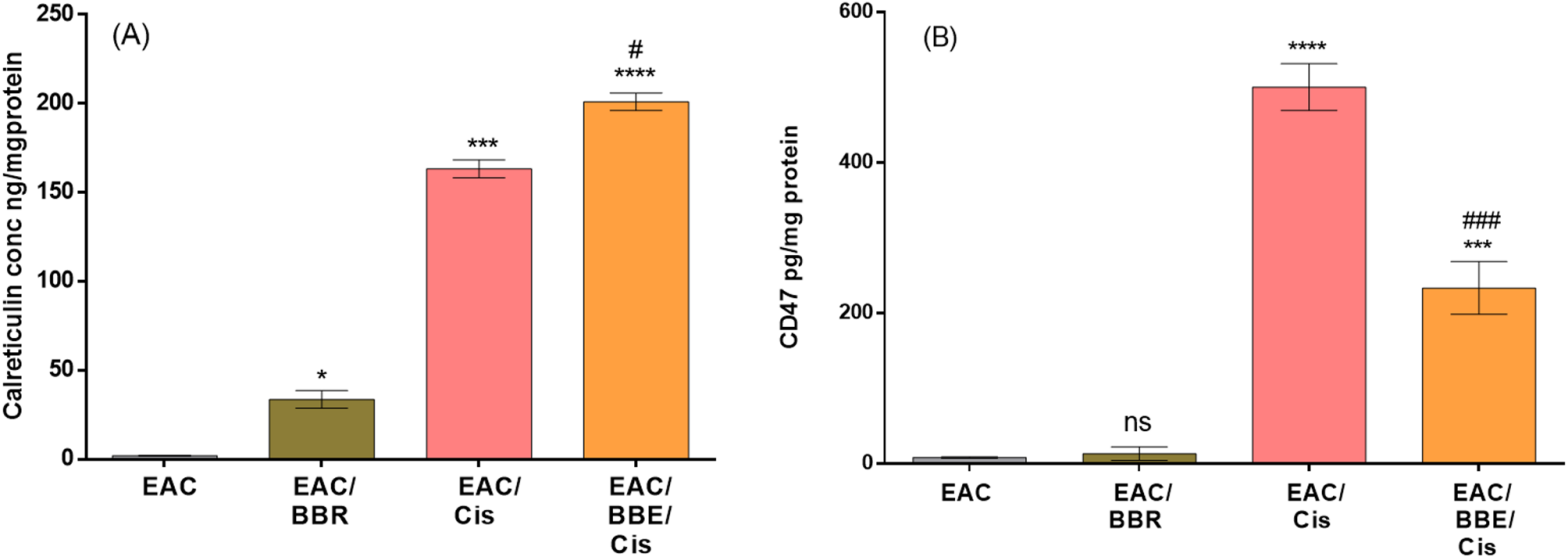
Efferocytosis biomarker changes: **(A)** Calreticulin, **(B)** CD47 concentrations. Results were expressed as mean ± SE, (*n* = 7). *p* < 0.05 is considered significant, where * compared with EAC group and # compared with EAC/cis group.

The results showed that treatment with BBR, Cis caused significant increase in CLR concentration with (*p* < 0.05) and (*p* < 0.001) respectively compared with EAC non-treated group, while combined treatment of Cis with BBR caused remarkable increase in CLR concentrations with (*p* < 0.05) when compared Cis alone treatment. On the other hand, CD47 showed significant increase in Cis with (*p* < 0.0001) when compared with EAC non-treated group, while combined treatment of Cis with BBR caused remarkable diminish in CD47 concentrations with (*p* < 0.01) compared with Cis alone treatment (Fig. 6A, B).

### Berberine enhanced apoptotic profile of cisplatin in EAC model

**Fig. 7:**
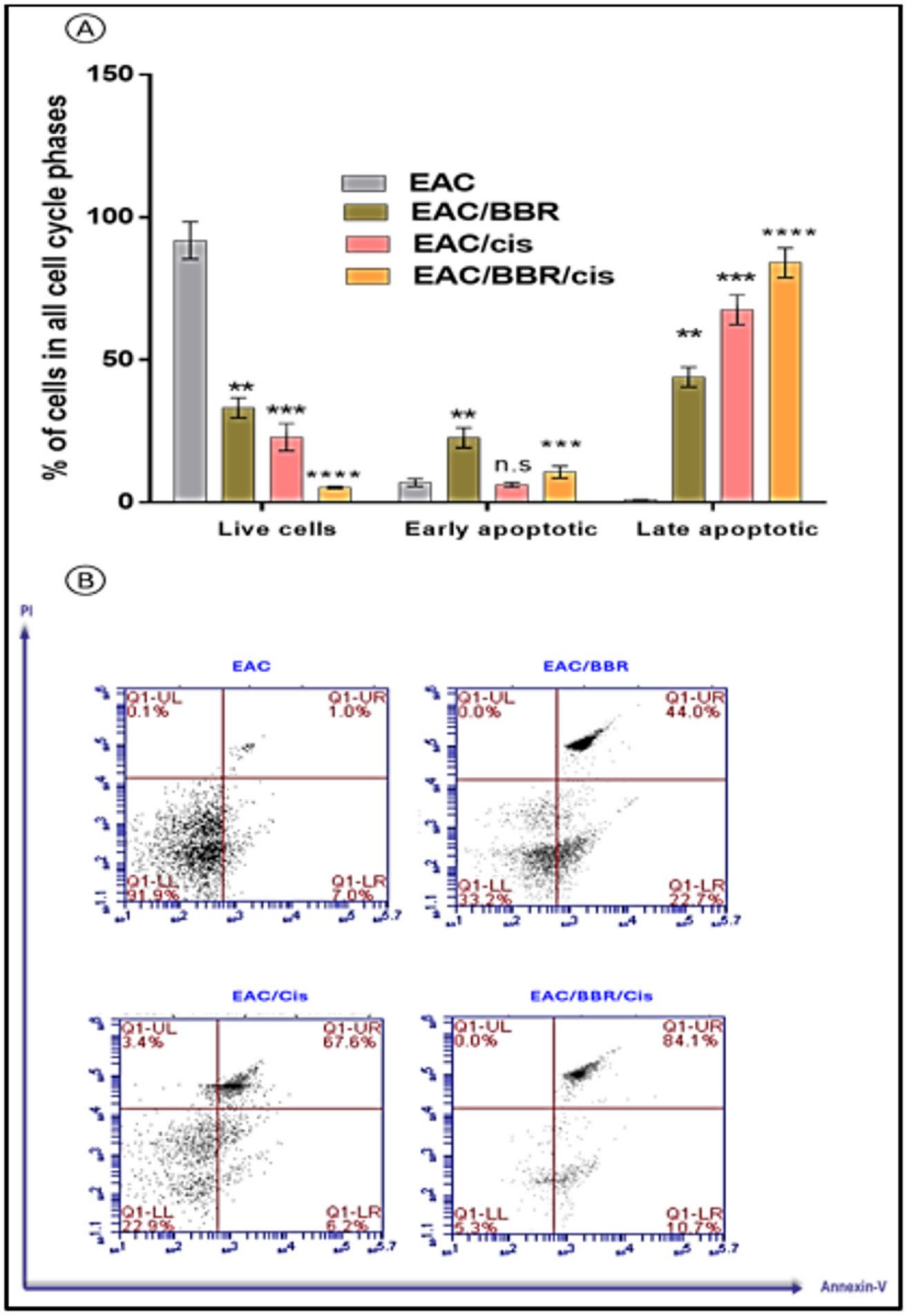
**(A)** Annexin V cell percentage and **(B)** dot plot of EAC-bearing mice group and all treated groups. Results were expressed as mean ± SE *n* = 7. Where ^*^ significant in all treated groups vs. EAC-bearing mice.

The results showed that BBR and Cis alone treatment showed significantly decreasing in live cells with values equal to 33.5% and 22.9% (*p* < 0.001) compared to EAC non-treated group. Additionally, combination treatment of Cis with BBR have the highest apoptotic effect as they showed significant decrease in live cell percentage with value equal to 5.3% (*p* < 0.0001) compared with EAC non-treated group. Moreover, treatment with BBR showed a high increase in percentage of early apoptotic cell 22.7% (*p* < 0.01) compared to EAC non-treated group, while Cis alone treatment or combined (BBR/Cis) showed no significant difference versus EAC non-treated group. Furthermore, treatment of EAC mice with BBR or Cis resulted in significantly increasing the percentage of late apoptotic cells by 44% (*p* < 0.01) and 67.6% (*p* < 0.001) respectively compared with EAC non-treated group. Additionally, BBR combination treatment with Cis elevated percentage of late apoptotic to equal to 84.1% (*p* < 0.0001) compared with EAC non-treated group. (Fig. 7)

### Berberine arrested G0/G1 phase cell cycle arrest of cisplatin in EAC model

**Fig. 8:**
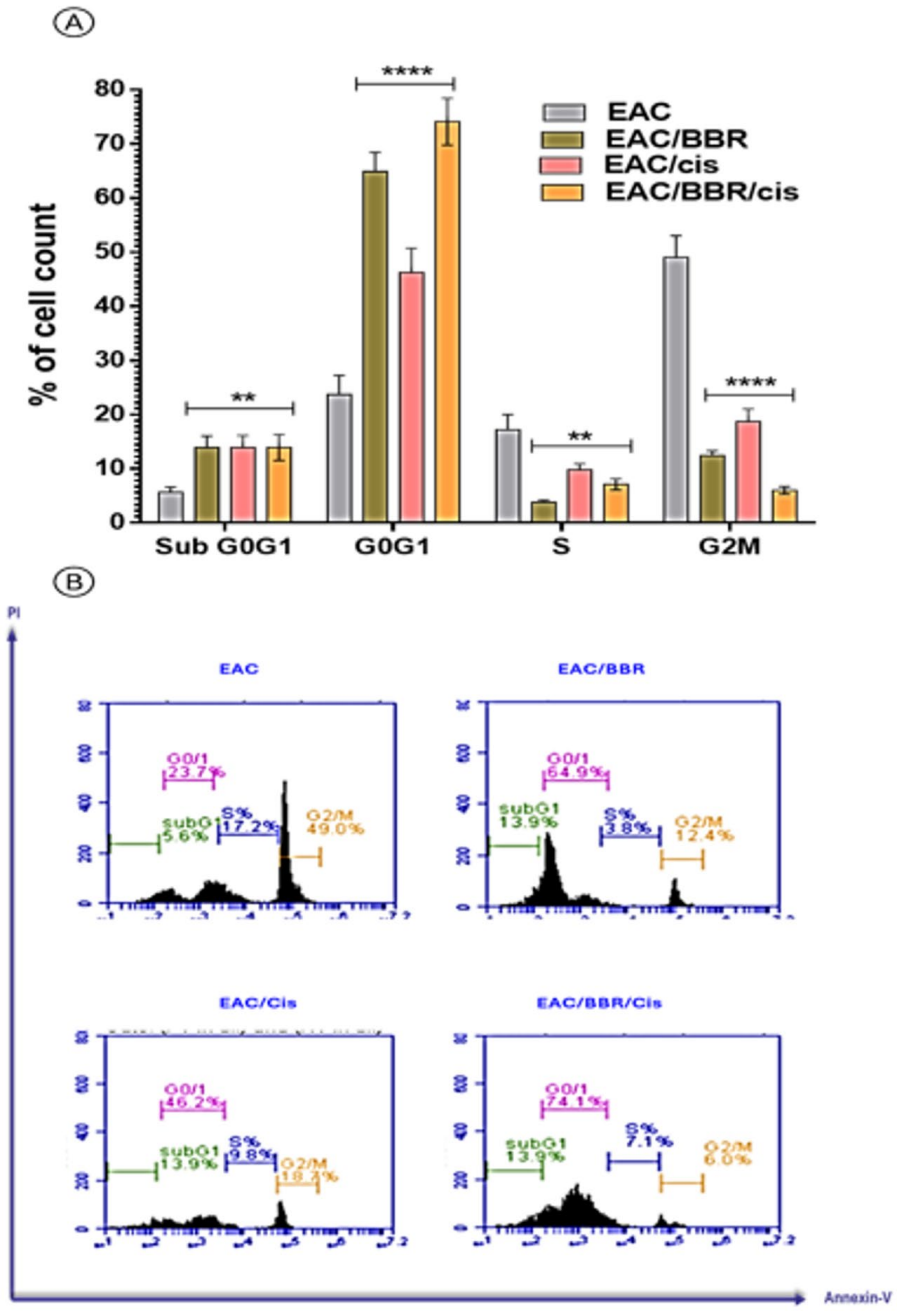
**(A)** Cell cycle arrest phases percentage and **(B)** chromatogram of all treated groups and EAC-bearing mice group. Results were expressed as mean ± SE *n* = 7. Where ^*^ significant in all treated groups vs. EAC-bearing mice.

All treated groups showed significant increase (*p* < 0.01) with arresting in G0/G1 phases more than the G2/M phases when compared with the EAC group, while combination treatment of Cis with BBR were the highest percentage of arresting in G0/G1 phases among other groups with value equal to 74.1% compared with EAC non-treated group. The S-phase in the treated groups with BBR, Cis alone or BBR combined with Cis has a significant decrease (*p* < 0.01) relative to the non-treated EAC group. Treatment with BBR, Cis showed a significant decrease in the percentage of the cells arrested in G2/M with (*p* < 0.001) when compared with EAC non-treated group, while BBR/Cis was the lowest value among groups which equal to 6.0% with (*p* < 0.001) when compared with EAC non-treated group. (Fig. 8)

### Akt1, Axl, Mertk and gas 6 expression declined in EAC cells by BBR

**Fig. 9:**
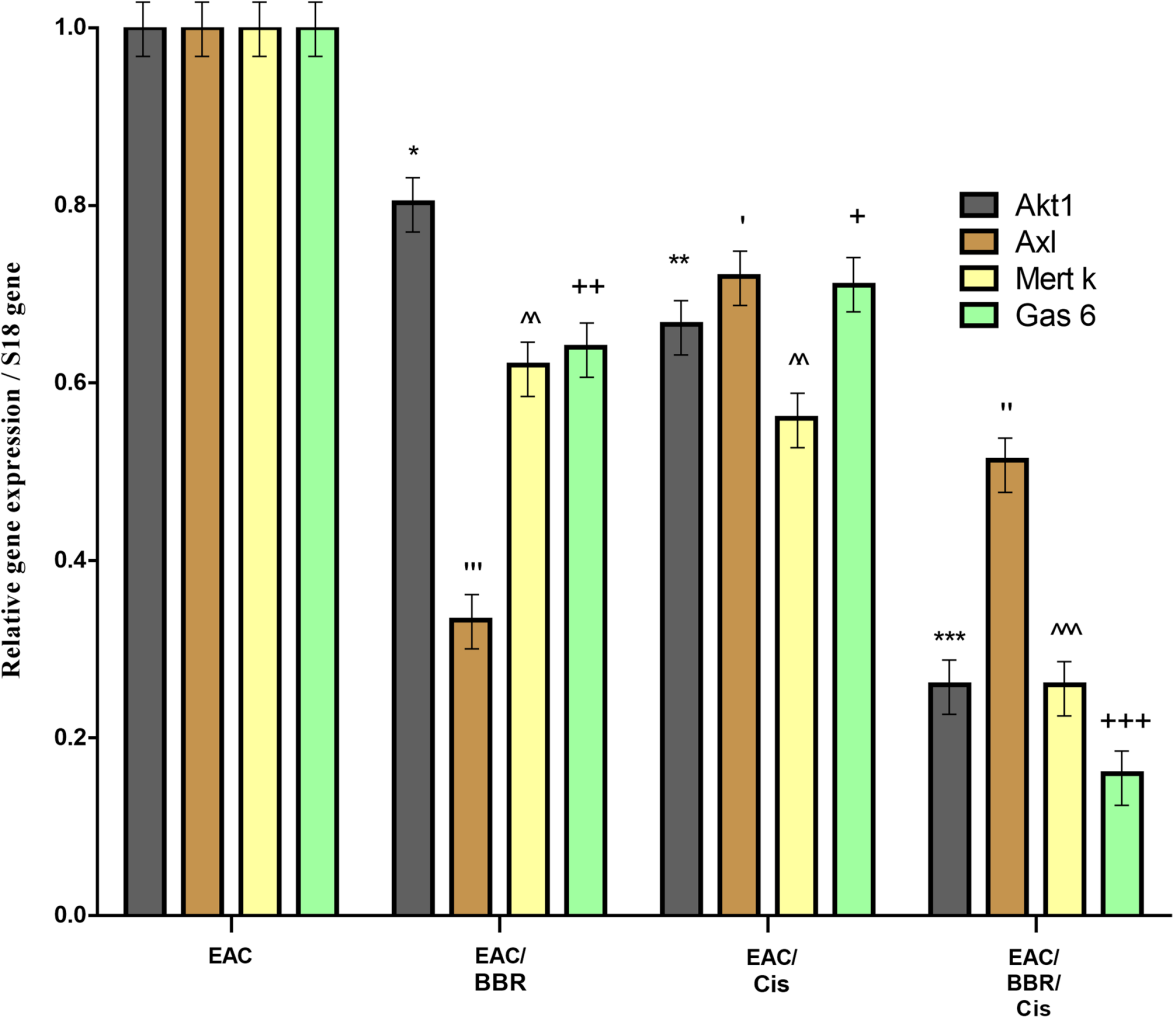
Relative expression of Akt1, Axl, Mertk and Gas 6 against S18 housekeeping gene in EAC untreated and all treated groups. (^*,‘,^,+^*p* < 0.0001) which significant to Akt1, Axl, Mertk and Gas 6 respectively for all treated groups vs. EAC-bearing group.

The results pointed out that Akt1was significantly down regulated in the mice treated with BBR (gpVI) with fold change equal to (0.8 ± 0.04) (*p* < 0.05), and in mice treated with (gp VII) (0.66 ± 0.07) (*p* < 0.001).On the other hand, Axl showed significant down regulation in BBR treated mice (gpVI) and cisplatin (gp VII) with fold change equal to (0.33 ± 0.064) (*p* < 0.001) and (0.723 ± 0.07) (*p* < 0.05) respectively. Mert k as well as Gas6 were significantly decreased (*p* < 0.05) in BBR treated mice (gpII) with fold change equal to (0.62 ± 0.064) and (0.64 ± 0.05), while treatment with cisplatin (gpVII) fold change were decrease to (0.566 ± 0.06) (*p* < 0.01) and (0.71 ± 0.64) (*p* < 0.05) respectively. BBR ameliorate cisplatin anticancer effect as observed in combination (gp VIII) which has the most minimal expression for all genes as shown in (Fig. 9).

### Berberine improves cisplatin liver pathological changes

**Fig. 10:**
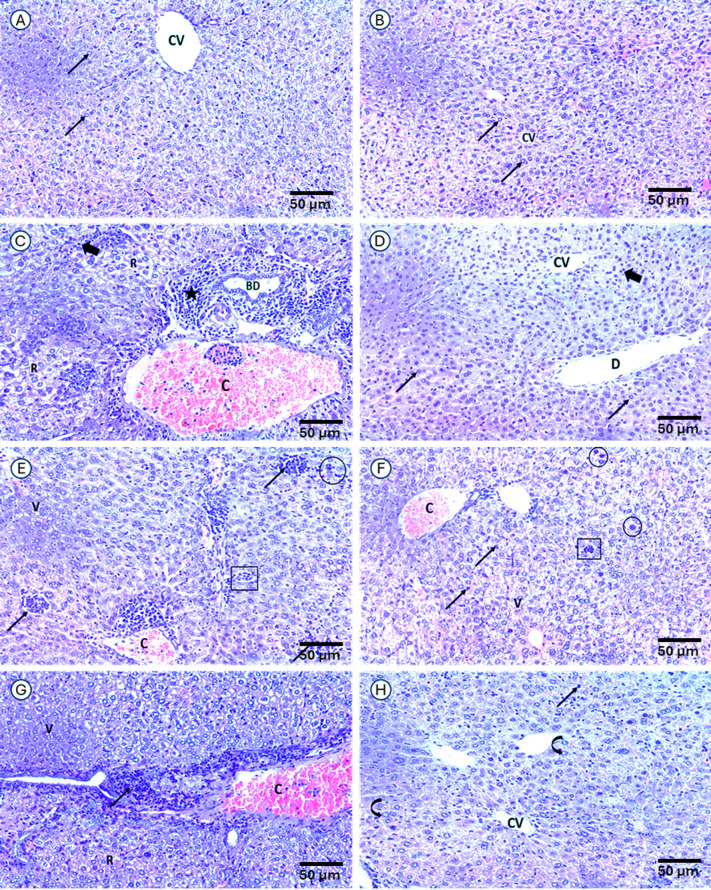
Histopathology of liver sections in all groups: Normal group **(A)**; Ctrl/BBE group **(B)**; Ctrl/Cis group **(C)**; Ctrl/BBE/Cis group **(D)**; EAC group **(E);** EAC/BBE group **(F)**; EAC/Cis group **(G);** EAC/BBE/Cis **(H)**. Where central vein (CV), normal binucleated hepatocytes with moderately acidophilic cytoplasm (thin arrow), pyknotic nuclei (thick arrow), rarefaction of cytoplasm of most of hepatocytes (R), inflammatory infiltration (*) and markedly congested blood vessel (C), dilatation of one central vein (D), pleomorphism, hyperchromatism and high N/C ratio, mitosis (anaphase) (circle) and tumor giant cell (square), (H&E Max. 200).

Normal group (GpI) liver architecture showed normal histological architecture consisting of normal hepatocytes with central rounded pale stained nucleus, central vein with radiating cords of hepatocytes surrounding it **(**Fig. 10A). Similar to it Ctrl/BBE (GpII) showed normal histological architecture more or less similar to control group consisting of normal hepatocytes with central rounded pale stained nucleus, central vein with radiating cords of hepatocytes surrounding it, indicating minimal toxicity (Fig. 10B). But Ctrl/Cis (GpIII) showed a disturbed hepatic architecture, pyknotic nuclei, rarefaction of cytoplasm of most of hepatocytes, inflammatory infiltration and markedly congested blood vessel in portal area, irregular and dilated bile duct in portal area (Fig. 10C). While Ctrl/BBE/Cis (GpIV) showed marked alterations indicating significant injury within normal histological architecture consisting of central vein with radiating cords of hepatocytes containing central round pale nuclei surrounding it except for dilatation of one central vein and few pyknotic nuclei (Fig. 10D). EAC-bearing group (GpV) showed severe damage with tumor infiltration by presence of undifferentiated, pleomorphic tumor cells that infiltrate and disrupt the liver architecture disturbed histological architecture, diffuse and focal infiltration with tumor cells, pleomorphism, hyperchromatism and high N/C ratio, congested central vein, cytoplasmic vacuolation of hepatocytes, mitosis (anaphase) and tumor giant cell, (Fig. 10E). As compared to EAC-bearing group (GpV), there was slight improvement of EAC/BBE (GpVI) in histological architecture, congested central vein, focal cytoplasmic vacuolation of few hepatocytes, few tumor cells, mitosis (prophase) and tumor giant cell, (Fig. 10F). While EAC/Cis (GpVII) showed disturbed histological architecture, area of rarefaction of cytoplasm and other showed vacuolations, markedly congested blood vessel, focal infiltration with tumor cells and tumor giant cell (Fig. 10G). In contrast, there was a marked improvement of histological architecture of liver in EAC/BBE/Cis which consisted of central vein and surrounding normal hepatocytes with pale stained nuclei and few scattered tumor cells (Fig. 10H).

## Discussion

The discovery of new anticancer drugs with high efficacy and broad safety profiles remains a significant challenge in cancer research. Current efforts are focused on modifying existing anticancer medications to overcome issues of toxicity and drug resistance. This study aimed to examine the effects of BBR, Cis, and their combination on EAC model, evaluating their impact on tumor growth, survival, organ function, oxidative stress markers, and cell cycle regulation.

Our results demonstrated that EAC mice treated with BBR, Cis, or their combination resulted in significant reductions in final body weight compared to untreated EAC groups, consistent with findings from previous studies^48,49^, which showed that in contrast to EAC group, cisplatin showed significant decrease in the final body weight as compared to the normal. Furthermore, our results matched with^50^ in which EAC treatment with hesperidin, cisplatin or their combination caused critical diminish in the final body weight as compared to EAC non treated group.

The combination therapy showed the most pronounced effects on tumor parameters, including decreased ascitic volume, reduced viable tumor cell count, and increased non-viable tumor cell count. These results align with other studies demonstrating cisplatin’s ability to reduce cell viability in cancer models^51,52^, whose reported that cisplatin combination reduced cell viability in two human small cell lung cancer cell lines and showed that BBR treatment sensitized the tumor cell to the cytotoxic effect of cisplatin by different mechanisms such as reducing the expression of drug transporters, enhancing apoptosis, and repressing PI3K/Akt/mTOR signaling. That confirm BBR ability to sensitize tumor cells to cisplatin’s cytotoxic effects through various mechanisms, including enhanced apoptosis and signaling pathway modulation, demonstrated by^53,54^.

The combination therapy also yielded the highest values for mean survival time (MST), percentage increase in life span (%ILS), and tumor growth inhibition (T/C%). These findings are supported by previous studies showing berberine’s ability to extend lifespan^55^ and cisplatin’s capacity to prolong survival in animal models^56^, who reported that the administration of Hesp and/or Cis to EAC-bearing mice resulted in notable increases in the MST and ILS%, and T/C % and prolonged the lifespan of animals by > 30% with respect to its control.

Liver and kidney function tests revealed that cisplatin treatment led to elevated serum levels of ALT and AST, while decreasing albumin and total protein levels. These changes indicate compromised liver function and are consistent with previous research^57^ that revealed that EAC inoculation in mice significantly increased the levels of serum (AST) and serum (ALT) and bilirubin, whereas levels of the serum total protein and albumin decreased significantly as compared to their levels in control group, suggesting compromised liver function. A pharmacokinetics study found that cisplatin can bind to human serum albumin (HSA), which is the most abundant plasma protein in serum^58^. Additionally, our finding complied with^59^. That reported that cisplatin can directly or indirectly (secondary to kidney damage) cause liver injuries and the increased plasma levels of AST and ALT indicate leakage of these enzymes from the cytosol of hepatic cells into the circulation system, which occurs after damage to liver cells or a change in the permeability of their membranes. All those results confirmed that berberine has protective capacities in liver diseases and protects against liver injury as reported in literature and therefore administration of berberine alone with cisplatin can guard from cisplatin induced hepatotoxicity^60,61^.

Similarly, cisplatin treatment increased serum urea and creatinine levels, indicating renal dysfunction. However, co-administration of berberine with cisplatin showed potential in alleviating cisplatin-induced nephrotoxicity, supported by previous studies demonstrating berberine’s renal protective properties^62,63^. Oxidative stress markers, including malondialdehyde (MDA), glutathione (GSH), catalase (CAT), and superoxide dismutase (SOD), were significantly altered in EAC and cisplatin-treated groups. The combination therapy with berberine and cisplatin showed improvements in these markers, suggesting berberine’s antioxidant properties help mitigate cisplatin-induced oxidative stress^62–64^. Furthermore^65^, have also shown that the hepatoprotection and nephroprotection afforded by berberine is mediated by stimulating levels and activities of numerous protective antioxidant enzymes and the concomitant reduction in lipoid peroxides. In different published studies, the EAC group exhibited a significantly decreased GSH, SOD (Aldayel et al., 2023), and significant decrease in GSH, SOD, CAT, and total thiols in the EAC group was also reported in^66^ study. This agrees with recent reports demonstrating the antioxidant properties of berberine and its role in restoring redox homeostasis^67–70^.

The combination therapy increased CLR concentrations while decreasing CD47 levels, potentially enhancing immune-mediated tumor cell clearance. These findings are supported by previous research on berberine’s ability to induce immunogenic cell death and downregulate CD47 expression^71,72^, in which treatment of SW620 cells (colorectal cancer) with berberine was anticipated to be ICD inducer and led to an increase in CRT on their cell surface that exposured of CRT, a protein that enables phagocytes to efficiently engulf dead cells observed in berberine-treated colon cancer cells^71^.

Gene expression analysis revealed downregulation of Akt1, AXL, GAS6, and MerTK genes in treated groups, consistent with previous studies on cisplatin and berberine’s effects on these signaling pathways^73–76^. That in those was reported a GAS6 expressions were significantly decreased under cisplatin treatment, showed the ability of cisplatin in reducing the Akt expression in colon cancer, and the expression of MerTK levels in NSCLC (non-small cell lung cancer) cell lines with a demonstration of BBR ability to induce autophagic cell death by inactivating the Akt/mTOR signaling pathway in melanoma cells and that berberine can be used as a possible target for the development of anti-melanoma drugs. These results are supported by recent molecular studies confirming that berberine suppresses PI3K/Akt signaling and related gene expression in cancer models^77^.

Cell cycle analysis showed that the combination treatment induced G0/G1 phase arrest and increased apoptosis more effectively than either agent alone. These results align with other studies demonstrating berberine’s ability to enhance cisplatin-induced cell cycle arrest and apoptosis^78–80^, which indicated an induction of G0/G1 apoptosis and cell cycle arrest by BBR/Cis. These findings are consistent also with recent studies showing that berberine enhances cisplatin sensitivity through modulation of apoptosis and PI3K/Akt signalling pathways^81^.

Histopathological examination revealed that cisplatin treatment caused significant liver damage, while the combination therapy with berberine showed reduced hepatic injury. This supports berberine’s potential hepatoprotective role when combined with cisplatin^82–85^, whose ddemonstrated that, a single dose of cisplatin was accompanied with the severe degenerative changes in the liver confirmed by the histopathological examination that exhibited local extensive necrosis of hepatocytes. Also showed that cisplatin injection caused hydropic degeneration in hepatocytes, coagulation necrosis and dilatation and hyperemia in the sinusoids. In addition to experimental evidence as in^86^, patented formulations of related compounds’ components have been developed, further supporting its translational potential as an anticancer agent^87,88^.

### Limitations and future perspectives

Conduct long-term studies on the treatment’s effects on cancer types and human cancer cell lines. Explore molecular mechanisms of BBE and Cis synergistic effects to pave the way for clinical translation. Furthermore, although protein expression was confirmed for key efferocytosis markers (CRT and CD47) via ELISA, the absence of protein-level validation for other molecular targets (Akt1, AXL, MerTK, and GAS6) represents a limitation of the current study. Nevertheless, the observed functional changes in efferocytosis, along with literature supporting mRNA-protein concordance for these genes, strengthen the reliability of the conclusions. Future studies will incorporate Western blotting or immunohistochemistry to comprehensively validate these findings.

## Conclusion

**Fig. 11:**
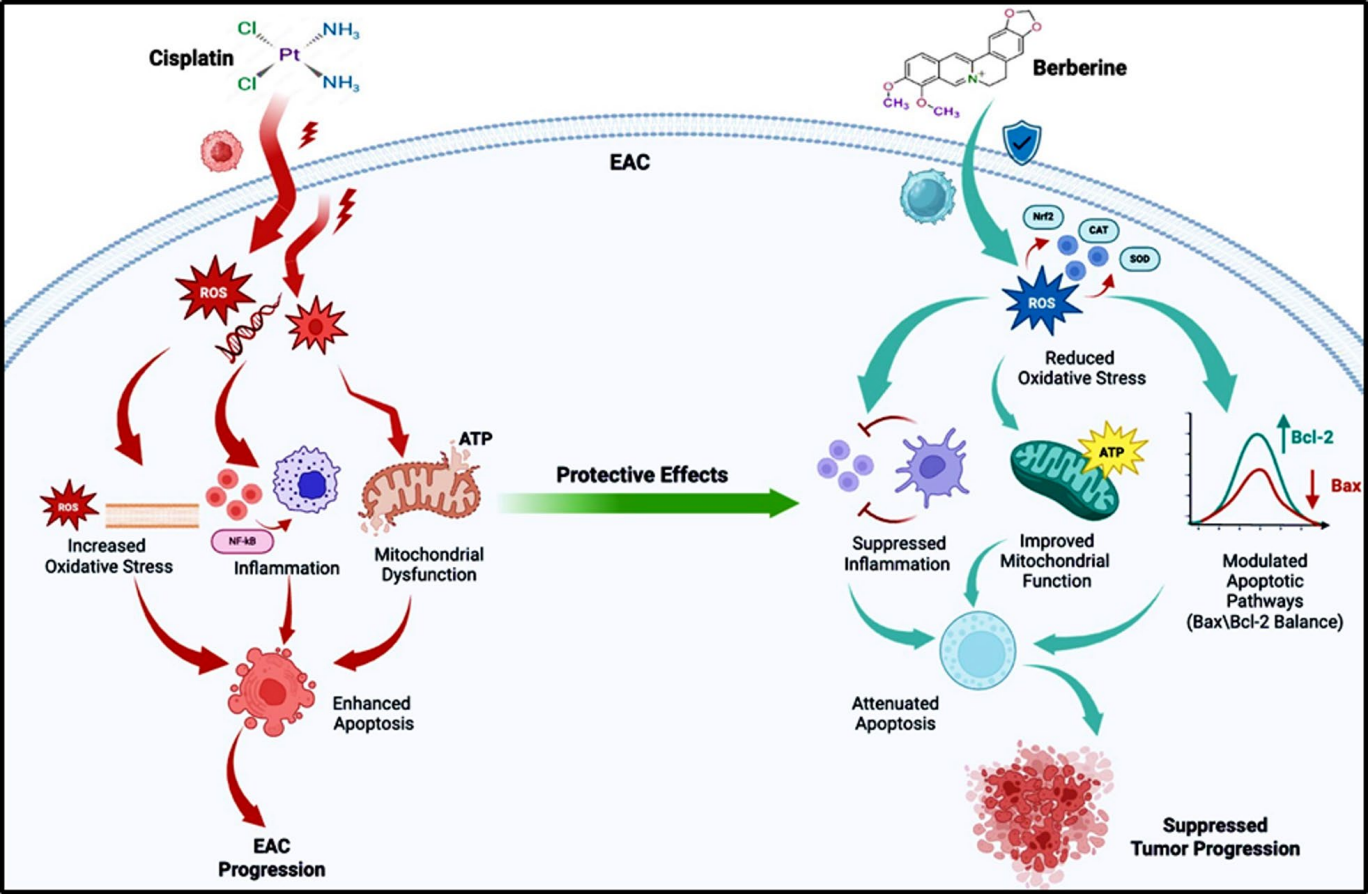
Proposed molecular mechanism of berberine in attenuating cisplatin-induced EAC progression. Cisplatin-induced tumor progression is associated with increased oxidative stress, inflammation, and mitochondrial dysfunction, leading to enhanced apoptosis. Berberine exerts protective effects by reducing oxidative stress, enhancing antioxidant defense systems, modulating apoptotic pathways (Bax/Bcl-2 balance), and improving mitochondrial function, ultimately suppressing tumor progression.

This study demonstrated BBR’s potential to enhance Cis anticancer efficacy while reducing toxicity in an EAC model. The BBR/Cis combination showed superior antitumor effects through reduced tumor volume, increased apoptosis, and improved survival. Berberine ameliorated cisplatin-induced hepatotoxicity and nephrotoxicity by modulating oxidative stress markers. The synergistic effects occurred via PI3K/Akt pathway downregulation and enhanced efferocytosis through calreticulin exposure and CD47 inhibition. These findings suggest BBR may be a promising adjuvant to Cis chemotherapy for improved tolerability as showed in Fig. 11.

## Data Availability

The data-sets used are available from the corresponding author on reasonable request.

## Abbreviations

Alb: Albumin
ALP: Alkaline phosphatase
ALT: Alanine transaminase
AST: Aspartate transaminase
BBR: Berberine
CAT: Catalase
Cis: Cisplatin
Create: Creatinine
CRT: Calreticulin
EAC: Ehrlich ascites carcinoma
ELISA: Enzyme-linked immunosorbent assay
GPx: Glutathione peroxidase
i.p.: Intraperitoneal
ILS: Increased life span
MDA: Malondialdehyde
MST: Mean survival time
PBS: Phosphate buffered saline
PI: Propidium iodide
PI3K: Phosphatidylinositol-3-kinase
qRT-PCR: Quantitative real-time polymerase chain reaction
RNA: Ribonucleic acid
T\C%: Tumor growth inhibition percentage
TP: Total protein
